# Preeclampsia – Will Orphan Drug Status Facilitate Innovative Biological Therapies?

**DOI:** 10.3389/fsurg.2015.00007

**Published:** 2015-02-26

**Authors:** Sinuhe Hahn

**Affiliations:** ^1^Laboratory for Prenatal Medicine, Department of Biomedicine, University Hospital Basel, Basel, Switzerland

**Keywords:** orphan disease, crowdfunding, advocacy groups, social networking, antithrombin, alpha-1-microglobulin, placental mesenchymal stem cells

## Abstract

It is generally accepted that the development of novel therapies to treat pregnancy-related disorders, such as preeclampsia, is hampered by the paucity of research funding. Hence, it is with great interest to become aware of at least three novel therapeutic approaches for the treatment of this disorder: exploiting either the anticoagulant activity of antithrombin, the free radical scavenging activity of alpha-1-microglobulin, or the regenerative capacity of placenta-derived mesenchymal stem cells. As these projects are being carried out by small biotech enterprises, the question arises of how they are able to fund such undertakings. A novel strategy adopted by two of these companies is that they successfully petitioned US and EU agencies in order that preeclampsia is accepted in the register of rare or orphan diseases. This provides a number of benefits including market exclusivity, assistance with clinical trials, and dedicated funding schemes. Other strategies to supplement meager research funds, especially to test novel approaches, could be crowdfunding, a venture that relies on intimate interaction with advocacy groups. In other words, preeclampsia meets Facebook. Perhaps similar strategies can be adopted to examine novel therapies targeting either the imbalance in pro- or anti-angiogenic growth factors, complement activation, reduced levels of placenta protein 13, or excessive neutrophil activation evident in preeclampsia.

## Introduction

Obstetrical concerns such as preterm labor (PTL), intra-uterine fetal growth restriction (IUGR), or preeclampsia (PE) remain huge concerns with regard to maternal or fetal morbidity or mortality (1–3). Indeed, prematurity has recently been listed as the leading cause of child death world-wide, accounting for almost 1 million of the 6.3 million children who die before the age of 5 (4, 5).

Although there has been considerable improvement in Western Europe, Canada, and the US to reduce maternal mortality associated with preeclampsia, it still accounts for one of the leading causes of maternal mortality during pregnancy (3). In developing countries, these rates are significantly higher, especially in cases with severe early onset PE (6) or due to the progression to “full-blown” eclampsia, where mortality rates can be as high as 15% (3). All in all it is a sad reflection on global health care that almost 800 women die daily as a result of pregnancy-associated complications (7), and it begs the question why efficacious intervention is so slow in forthcoming or implementation (8).

## Why the Progress in the Development of Therapeutics in Feto-Maternal Medicine so Slow?

Although the health care burden by pregnancy-related conditions, specifically PE, is considerable with an estimated global health care cost of $3 billion, it is dwarfed by the incidence of cancer, which is approximately 28 times greater (8.2 million deaths per annum) (9). A further concern that could hinder the active participation of pharmaceutical industries in the development of new drugs for prenatal use is the disaster experienced with thalidomide, due to its unexpected teratogenic effect (10). It is of interest that the underlying mechanism has only recently elucidated in 2014, several decades later (11). A further issue is that the relationship between patient and physician in the Internet age is complicated by the reliance of the former on untrustworthy data or unfounded skepticism of new untried developments (12, 13).

Consequently, the argument by pharmaceutical companies, or diverse research funding agencies to focus their resources on more pressing concerns, may at first hand appear rational. However, closer scrutiny indicates that global R&D funding for pregnancy-associated complications is grossly underfunded, either by the private sector or national bodies, with less than 1% or the health research budget in the US or UK being allocated to the topic of reproductive health (14, 15). This indicates that pressing obstetrical concerns such as PE have been related to the status of “orphan diseases” or less, and consequently is listed as such by the Food and Drag Administration (FDA) and European Commission (16).

Such scientific relegation has, however, led to an almost complete stifling of the development of new therapeutic agents for use in pregnancy, with the result that physicians are increasingly obliged to resort to “off-label” use of drugs for which no safety indication is available (17–19).

## Does Being Labeled an Orphan Disease have any Benefits?

It is unclear why PE could be listed as an “orphan or rare disease” as the defining criteria for such status is an incidence of less than 5 in 10,000 individuals, granted that PE affects between 3 and 8% of all pregnancies (20). In the context of the entire population, however, the number of people affected by PE is quite small, and has been estimated to be of the order of 3.8 per 10,000, thereby comfortably securing a slot in the rare disease catalog (16).

A driving force behind the US Orphan Drug Act (ODA) of 1983 and similar European, Japanese, Australian, or Singaporean regulations was the decision that “patients suffering from rare conditions should be entitled to the same quality of treatment as other patients” (21). The main aim of such legislation is to provide specific incentives to promote drug development for rare conditions. These include market exclusivity, reduction in regulatory fees or subsidies (tax breaks) with clinical trials (21). In addition, special funding schemes have been developed by the NIH (Office of Rare Diseases Research), the EU, and other institutions (NORD: National Organization for Rare Disorders), specifically targeting rare conditions (22). Successful funding, however, may require lobbying or engagement by advocacy groups (23). Consequently, successful funding may in future require considerable political action and mobilization of interest groups.

## Is Crowdfunding the Solution?

If scientific success in future may depend on a meaningful interaction with advocacy groups, it appears obvious that this scenario may be exploited to secure necessary financial resources by means of crowdfunding. A development of social media and globally linked communities, crowdfunding, is a method of raising venture funds from a large number of individuals via the Internet (24–28). To date most crowdfunding approaches have been used in the arts or technology development, but scientific usage is increasing rapidly.

An examination of 159 crowdfunding attempts using the #Scifund platform indicated that success largely depended on the degree of interaction between scientists and their audience, and the ability to expand this network effectively (24). Although the turn-over generated by the international crowdfunding industry is proposed to be greater than $1 billion, with more than $200 million being raised via a single website, the rewards for the scientific community have been less spectacular, with the average amount secured estimated to be less than $50,000 (24, 26, 27).

Nevertheless, crowdfunding may play an important role in assisting the establishment of academic spin-off biotech companies geared toward the development of innovative therapies (25). The incentive for crowdfunding participants in this instance is the feature of equity and the possibility of a very high rate of return.

## Are any Innovative Biological Therapies Being Developed?

In view of the largely negative preamble in this mini-review, it is gratifying to note that at least three innovative biological therapeutic strategies are being pursued for the treatment of PE. It is also noteworthy that all three are being carried out by small newly established companies, solidifying the suspicion that PE does is not an interesting feature for large pharmaceutical companies.

The first of these small companies is Revo Biologics Inc., which is investigating the use of recombinant antithrombin (rAT), termed ATryn^®^, as an Investigational New Drug (IND) for the treatment of preeclampsia in pregnant women. Their goal is not a full cure but rather to prolong gestation, thereby decreasing fetal or neonatal morbidity or mortality (29).

The rationale behind the approach of Revo Biologics Inc., is that preeclampsia is associated with a pro-thrombotic condition (30), and that previous use of anti-coagulants, such as antithrombin, has been shown to have some benefit (29, 30). This includes at least two prospective case-control or feasibility studies conducted in Italy and Japan (31–33).

Enrollment to test ATryn^®^ is underway in a Phase 3 clinical trial termed Preserve-1 (Prospective Randomized Evaluation of the Safety and Efficacy of Recombinant Antithrombin in Very Preterm Preeclampsia) (29).

A second approach is being explored by A1M Pharma AB in Sweden, a biotech spin-off from Lund University, which focuses on the finding by the founders that alpha-1-microglobulin (A1M) can act as a scavenger and remove toxic free radicles (34). By the use of a sheep model for PE, which is induced by starvation-mediated hemolysis, it was shown that exogenous A1M application lead to significant amelioration, detected by an examination of placental and kidney pathology (35). It is unclear if any clinical trials have been launched or are planned to test A1M application in human patients. Of interest is that A1M Pharma successfully lobbied the European Medicines Agency (EMA) Committee for Orphan Medicinal Products (COMP) in order to secure an Orphan Drug status for preeclampsia.

A third very intriguing development is that pursued by the Israeli company Pluristem Therapeutics Inc., which has a series of proprietary technologies focused on the isolation and expansion of mesenchymal-like stem cells from the placenta (PLX) (36). The use of these PLX cells has been explored for the treatment of ischemia or to improve the engraftment of cord blood hemopoietic stem cells (37–39). It now appears that Pluristem is considering the use of these PLX cells for the treatment of preeclampsia, and is following a similar strategy to that of A1M Pharma, in that it has submitted a FDA Orphan Drug application for PE (40).

## Could Other Strategies be Explored for the Development of Novel Biological Therapies?

The past decade has seen a huge expansion in our knowledge of the underlying etiology involved the development of PE (41). These include the discovery of an imbalance in the pro-angiogenic cytokine PIGF (Placenta Growth Factor) and the anti-angiogenic factor sFlt-1 (soluble fms-like tyrosine kinase 1) in cases at-risk for PE, and the examination of how this anomaly to contributes to endothelial dysfunction (42). Additional discoveries include the contribution of complement dysregulation to placental damage (43), the possible involvement of placental galectins such as PP13 (placental protein 13) in placental development and immune modulation (44), or the excessive occurrence of neutrophil extracellular traps (NETs) in preeclamptic placentae (45).

Of these the elevated presence of the anti-angiogenic factors sFlt-1 would seem to be a prime target, as it has previously been shown that extracorporeal removal of this factor by apheresis can extend the pregnancy of women with manifest early onset PE by as much as 23 days (46). An alternative may be to target complement dysregulation, since treatment with a specific inhibitor (eculizumab) has been shown to significantly prolong gestation in a case with PE/HELLP syndrome, thereby improving neonatal outcome (47).

Treatment with recombinant PP13 may be an attractive option in future, granted that its application in pregnant rats is suggested to reduce blood pressure (44). The involvement of excessive NETosis in the etiology of auto-inflammatory conditions such as rheumatoid arthritis (48, 49) or systemic lupus erythematosus (SLE) (50, 51) opens up the possibility that novel therapeutics will be developed to counter this undesired neutrophil activity (52). This could pave the way for possible use in PE.

## What is the Way Forward?

The low level of funding in reproductive medicine is a challenge, which need to be overcome if effective therapies are to be developed for the numerous concerns which continue to beset obstetrical practice. On the one hand this may rely on innovative strategies such as crowdfunding or to somewhat subversive methods, such as applying for orphan disease status. The success of either will depend largely on adequate lobbying, which implies that much closer ties with advocacy groups using social networks will become the order of the day.

## Concluding Remarks

In this review the focus was on three small companies who are driving the development of new therapeutic strategies for the treatment of PE, which makes it clear that innovation does not rely on huge funding bodies or multinational pharmaceutical companies. Rather, like David when facing Goliath, it relies on flexibility, nimbleness of feet, and a vision or calling to make a significant contribution to the betterment of humankind.

## Conflict of Interest Statement

The author declares that the research was conducted in the absence of any commercial or financial relationships that could be construed as a potential conflict of interest.

## Abbreviations

A1M, alpha-1-microglobulin; COMP, Committee for Orphan Medicinal Products; EMA, European Medicines Agency; FDA, Food and Drug Administration; IND, investigational new drug; IUGR, intra-uterine fetal growth restriction; ODA, Orphan Drug Act; NORD, National Organization for Rare Disorders; PE, preeclampsia; PIFG, placenta growth factor; PLX, mesenchymal-like stem cells from the placenta; PP13, placental protein 13; PTL, preterm labor; rAT, recombinant antithrombin; sFlt-1, soluble fms-like tyrosine kinase-1; VEGF, vascular endothelial growth factor.
